# Thumb performance of elderly users on smartphone touchscreen

**DOI:** 10.1186/s40064-016-2877-y

**Published:** 2016-07-29

**Authors:** Jinghong Xiong, Satoshi Muraki

**Affiliations:** 1 Graduate School of Design, Kyushu University, 4-9-1 Shiobaru, Minami-ku, Fukuoka City, 815 8540 Japan; 2 Faculty of Design, Kyushu University, 4-9-1 Shiobaru, Minami-ku, Fukuoka City, 815 8540 Japan

**Keywords:** Smartphone touchscreen, Thumb, Elderly, EMG, Perceived exertion

## Abstract

This study investigated the relationship between thumb muscle activity and thumb operating tasks on a smartphone touchscreen in elderly users (right hand posture). Three thumb muscles were targeted in the experiment, namely, abductor pollicis brevis, abductor pollicis longus (APL) and first dorsal interosseous (FDI). The results showed that the elderly participants developed fatigue rapidly and tapped more slowly when operating on smaller buttons (diameter 3.0 mm compared with 9.0 mm) and moving in the flexion–extension (compared with adduction–abduction) orientation. Meanwhile, electromyography and perceived exertion evaluation revealed significant increases in FDI in the small button task, and results for APL were significantly greater in the flexion–extension task. This study suggests that the use of small touch-buttons and flexion–extension movement should be minimised in the handheld touchscreen interface design for elderly users.

## Background

As one of the fastest growing technologies in modern times, the astonishing growth of phone-owning population seems to be continuing for all age groups and regions around the world, irrespective of the economic disparities (Boretos 2007). By 2011, the mobile phone ownership globally hit the 6 billion mark (International Telecommunication Union 2012). Meanwhile, with the rapid development of computer and telecom technologies, mobile phones have been becoming much smarter both in size and in function in recent years. In particular, in the form of smartphones, which are embedded with touchscreens and offer personal computer-like functions and more user-friendly interfaces compared with tactile keypad mobile phones, this new generation of mobile phones is expected to generate even more users in the near future, including among the elderly. However, with the increase of aging populations across over the world, whether or not elderly users can keep pace with the rapid progress of mobile technologies is a challenge for the mobile interface designers and ergonomists.

When using a touchscreen smartphone, elderly users are likely to benefit from some special interface features. For instance, in order to enhance the ability to recognise touch targets, many elderly users may prefer to set the fonts and icons to a larger size. However, this could increase the time that they require to flip through the menus, and their thumbs (when operating with one hand) have to pass over a larger area to reach the larger targets. By doing this, the likelihood of making input errors and dropping the phone may increase, consequently increasing the physical and mental effort required to use the touchscreen smartphone. Moreover, from a kinematical perspective, the difficulties for elderly users in operating a touchscreen smartphone seem severe. A study found that small touch button size, poor spacing among the touch buttons and inconvenient location of targets on touchscreen smartphones significantly reduced the finger pointing performance in elderly users (Hwangbo et al. 2013). In addition, Cole and Rotella (2002) reported that aging is a significant factor that impairs visual cues for predictive control of finger force when gripping a mobile phone in hand. Olafsdottir et al. (2007) also pointed out that decreased performance in accurate multi-finger movements normally accompanies old age. Moreover, it was found that aging has a degenerative effect on hand functions, especially in precision grip, pinch force and maintaining a steady pinch posture (Carmeli et al. 2003; Ranganathan et al. 2001). Muraki et al. (2010) revealed that the elderly tended to have more pushing errors on small cell phones than young participants. Another study also showed that middle-aged subjects (40–65 years) exhibited significantly slower input speed than the young (20–32 years) when using a touchpad device (Armbruster et al. 2007). More significantly, Xiong and Muraki (2014) found that tapping small touch buttons on a smartphone touchscreen tends to increase the muscle effort of the first dorsal interosseous (FDI) in young participants, which was regarded as the main reason for reduced input thumb performance on a touchscreen.

Therefore, elderly people may adopt different operating postures compared with younger users in order to overcome those difficulties caused by aging. For example, when the phone is operated by the right hand, elderly users may have reduced capability to make their thumb reach the upper-left area of the screen surface, and the muscular effort and subjective exertion in the thumb could differ from those in younger users. As a result, elderly users may have a limited operating area regardless of any increase in screen size (Xiong and Muraki 2016). Thus, through the assessment of muscle activities and perceived exertion in three aspects on a smartphone’s touchscreen, namely, button size (larger: 9.0 mm in diameter, small: 3.0 mm), thumb movement orientations (adduction–abduction and flexion–extension) and thumb circling directions (clockwise and counter-clockwise), the present study assumed that these three aspects would have significant effects on the elderly users’ thumb performance when operating the device. Based on an improved understanding of the effects of these three aspects, it is believed that some useful design suggestions for better handheld touchscreen device user interfaces specifically for elderly users could be gained.

## Methods

The method of the present study replicated that in a previous study reported by Xiong and Muraki (2014). The previous study clearly explained the relationship between thumb muscle effort and experimental tasks on smartphone touchscreens. However, the measures of previous study merely focused on young participants (age 24.5 ± 2.2), therefore the discussion of results may have been limited to explain for young adults. In order to retain the consistent reliability of study results for elderly participants, the present study adopted the identical method retrieved from the previous study. In addition, the task settings of both two studies referred to those in previous studies reported by Trudeau et al. (2012a, b, 2014), and hence some similarities in the experimental task settings among these studies can be found in the present study.

### Participants

The study recruited a total of 20 right-handed senior citizens (10 males, 10 females, mean ± standard deviation [SD] age 65.5 ± 1.5 years). We measured the dimensions of the right hand to compare with those in the referred study reported by Xiong and Muraki (2014). The hand length (tip of middle finger to bottom edge of palm), hand breadth (outside edge at metacarpo-phalangeal joint of index finger to that of little finger) and thumb length (tip of thumb to apophysis at proximal end of metacarpal) of elderly participants were 176.0 ± 13.1, 80.1 ± 4.6 and 101.6 ± 8.3 mm respectively. The statistics did not reveal significant differences between the two studies (referred study: 180.3 ± 16.7, 82.0 ± 9.1 and 101.2 ± 9.9 mm). Participants reported no impaired vision, none of musculoskeletal disorders or any motor symptoms. This study was approved by the Institutional Ethics Review Board of Kyushu University, Japan, and informed consent was obtained from all participants.

The participants were allowed about 10 min to familiarise themselves with the experimental phone mock-up, and accordingly decided on a holding posture that enabled them to hold the phone mock-up comfortably and steadily for the experimental tasks. Although no participant owned a touchscreen smartphone, they had been using push-button mobiles for over 10 years in daily use, which means that the participants were familiar with small hand-held devices. In the 6 months prior to the experiment, they have tried using touchscreen smartphones several times when considering switching to touchscreen phones. Thus all participants claimed to have sufficient knowledge and confidence to carry out the experimental tasks without distortions caused by unfamiliarity with touchscreen smartphones.

### Protocol

**Fig. 1:**
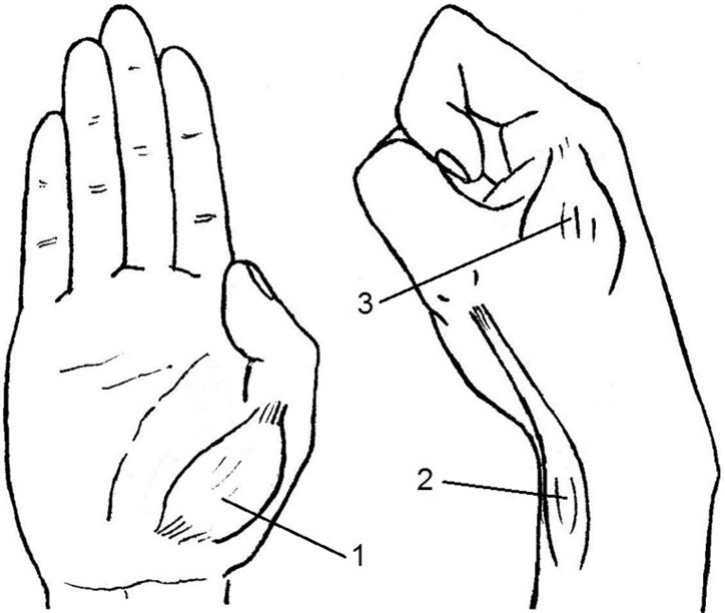
The three targeted thumb muscles. *1* *APB* abductor pollicis brevis. *2* *APL* abductor pollicis longus. *3* *FDI* first dorsal interosseous

Three muscles in the right thumb were selected (Fig. 1), namely, abductor pollicis brevis (APB), abductor pollicis longus (APL) and first dorsal interosseous (FDI). The study used a physical copy of iPhone4 for the participants to undertake the experimental tasks. The phone mock-up dimensioned at 115.2 × 58.5 × 9.3 mm, with a weight of 140.0 g. The tested area on the phone mock-up also was a copy from the iPhone4, which was the keyboard layout (sized at 50.0 × 33.0 mm, distanced 20.0 mm from the bottom with central alignment). Participants seated in front of a desk (height: 70.0 cm) comfortably, the height of chair (armless) was adjustable that matched various body heights. In order to ensure the participants could remain their concentrations on the experimental tasks, the participants were asked to place their arms on the desk in a comfortable posture based on their preference. In addition, the participants were allowed to shift the postures during the experiment, as long as the arms and wrists were fully supported by the desk, and the phone holding posture would not be affected. Furthermore, the temperature and other environmental conditions were well controlled throughout the experiment for providing optimal experimental environments.

### Tasks

 The experimental task asked the participants to tap the round plastics touch buttons on the tested area of the phone mock-up by their thumbs (right hand posture). The buttons were extremely thin pressure sensor (FlexiForce A201-1, Nitta, Japan) firmly attached on the screen surface. Participants began performing the task by a start signal, and they were asked to stop at any point when fatigue was perceived in the thumb (Table 1).

**Table 1:** Experiment outline

*Tapping*	
Large button	Fixed speed
	Max speed
Small button	Fixed speed
	Max speed
*Moving*	
Ad–Abduction	Fixed speed
	Max speed
Flexion–extension	Fixed speed
	Max speed
*Circling*	
Clockwise	Fixed speed
	Max speed
Counter-clockwise	Fixed speed
	Max speed

**Fig. 2:**
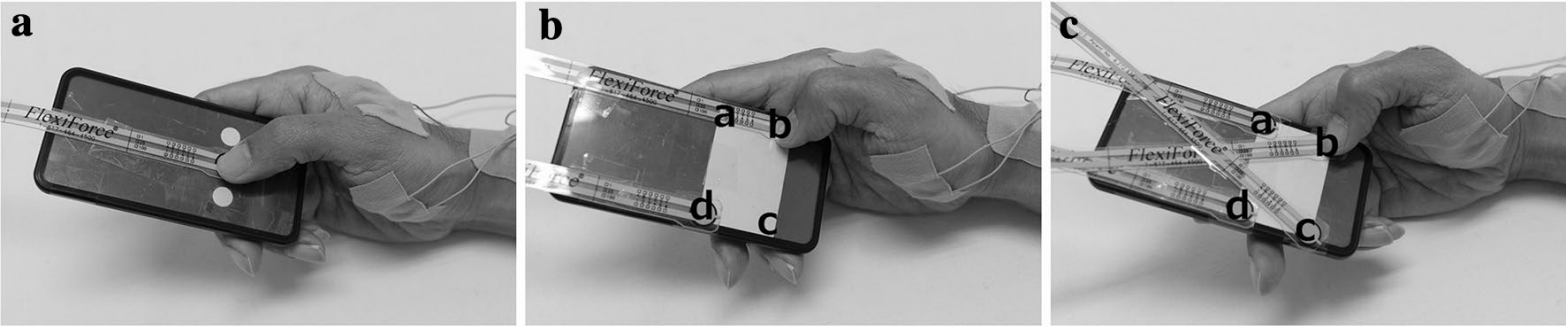
Three tasks in the experiment. **a** Tapping task: large button task—button diameter of 9.0 mm, small button task—3.0 mm. **b** Moving task: ad–abduction task—orientation between *a* and *c*, flexion–extension task—*b* and* d*. **c** Circling task: clockwise task—direction *a*–*b*–*c*–* d*, counter-clockwise task: *a*–*d*–*c*–*b*

The experiment was divided into three sections as tapping, moving and circling sections. Tapping section required the participants to tap a button in the physical centre of the tested area (Fig. 2a). The diameter of button was 9.0 mm in the larger button task, and it was reduced to 3.0 mm in the small button task. Moving section included two tasks, namely, adduction–abduction and flexion–extension tasks. For adduction–abduction task, the participants tapped between two pressure sensors (diameter: 9.0 mm) at the two adduction–abduction orientation corners in the tested area, whereas they tapped the other two corners in the flexion–extension orientation for the flexion–extension task (Fig. 2b). Circling section asked participants performed clockwise and counter-clockwise tasks. In the circling section, the participants were asked to tap on the four pressure sensors (diameter: 9.0 mm) of the corners in the tested area (Fig. 2c). As the task name suggested, the tapping direction between the tasks was opposite. In the clockwise task, the tapping direction was from top-right, bottom-right, bottom-left and then top-left. Both the tasks started from top-right button.

Each section comprised two tasks. In the tapping section, the tasks were “large button task” and “small button task”; in the moving section, the tasks were “ad–abduction task” and “flexion–extension task”; and in the circling section, the tasks were “clockwise task” and “counter-clockwise task”. In each task, participants performed two subtasks, namely, fixed speed and max speed subtasks. In the fixed speed subtask, participants performed one tap per second in time with a digital metronome (frequency: 1 Hz). In the max speed subtask, the participants were asked to tap the buttons as quick as they possibly could. After the participants completed each subtask, a 5-min rest was provided until any feeling of fatigue had passed, before the next subtask. The order of tasks was randomised.

### Measures

This study defined that how quick the participants tapped on a button in the max speed subtask as the “tapping speed”. The taps were counted by the pressure sensors that were connected to a pressure wave record device. It was defined that the time from the point of starting tapping on the buttons to the point of stopping was the “fatigue time”. The point that the participants started perceiving the “fatigue” in the targeted muscles that caused them stopping the tapping actions was defined as the time of stopping. The perceived exertion evaluation asked the participants to rate their “fatigue” for each tested muscle. Immediately after each task was finished, the participants rated filled out a Borg’s CR10 form for the evaluation. In this form, mark 0 stands for no feeling of fatigue at all whereas mark 10 means for the maximum level of fatigue.

Prior to the experiment, the participants were given instructions about the three targets of thumb muscles (Fig. 1), including the positions, physical and functional features. In the present study, “fatigue” was defined as either a fatigued sensation or uncomfortable feelings in these three muscles that could drive them to stop continuing the experimental tasks. The number of targeted muscles was relatively small, and the hand dimensions of the participants showed little difference, so it was easy enough for the participants to distinguish “fatigue” from other unrelated feelings and to maintain similar measuring standards. All participants claimed that they fully understood what to do for the experiment. Therefore, we believe that the definition of “fatigue time” and the perceived exertion evaluation were reliable for the study.

All the tested muscles were registered for EMG assessment. For APB, the electrodes were attached on the belly of the muscle between the metacarpophalangeal and carpometacarpal joints (Seror et al. 2011). The electrodes of APL were placed on the forearm proximal to the styloid process of the radius. As for FDI, electrode attachment was at the muscle belly above the thumb base and proximal to the index finger base (Gustafsson et al. 2011). An EMG device (SYNA ACT, MT11, NEC, Japan) and program Vital Recorder II (Kissei Comtec, Japan) were used to record the real time muscle activities (sensitivity: 1000 μν, sampling frequency: 1 kHz, time constant: 0.03 s, high-frequency filter: 100 Hz). After all raw data was collected, a program KineAnalyzer (Kissei Comtec, Japan) was used for filtration and analysis. This program filtered the full waves of raw EMG signals at low-pass 20 Hz and notch 60 Hz. In order to represent the muscle activities, the data analysis selected integrated EMG (iEMG), contraction time and iEMG/s were selected as the indexes. The iEMG (mV s) is the absolute integral of the raw signal within a contraction, and contraction time means the time (seconds) that a thumb muscle contracts for each tapping action. Thus, the iEMG/s (mV) is the absolute integral divided by the contraction time (Xiong and Muraki 2014). The tapping action was determined by the waves of tapping pressure. The wave range from the point that a wave begins to the beginning point of the next wave.

The data presentation is referred to the percentages of reference values. Firstly, each tapping action’s iEMG, contraction time and iEMG/s were calculated and then averaged. Secondly, for each participant in a task, the 50th percentiles of these three indexes were calculated, and then the values of iEMG, contraction time and iEMG/s were divided by their 50th percentiles to calculate the individual the percentages of reference value. The final percentage of reference value (Xiong and Muraki 2014) was the mean value across all the participants with variability of standard error (SE). The present study involved 50th percentiles rather than percentage of Maximum Voluntary Contraction (%MVC). This was based on the concern that maximal voluntary isometric exertion might increase the risk of damage to the small muscles of the thumb; and such damages could be extremely painful and hard for elderly subjects to recover from. In order to minimise the risk, the non-normalised EMG was chosen, and the data were presented as 50th percentiles rather than absolute values to reduce the bias caused by individual differences.

### Statistics

The paired *t* test was used for calculating the differences in fatigue time, tapping speed and perceived exertion evaluation between tasks; and Wilcoxon signed-rank test was used for calculating the differences in the actual values of iEMG/s, contraction time and iEMG between tasks. Two-way ANOVA was applied to examine the influences of muscles and tasks. All tests were conducted using IBM SPSS Statistics Version 20.0.0 (Japanese language package) and the statistical significance was accepted at p-values less than 0.05.

## Results

### Tapping section

**Fig. 3:**
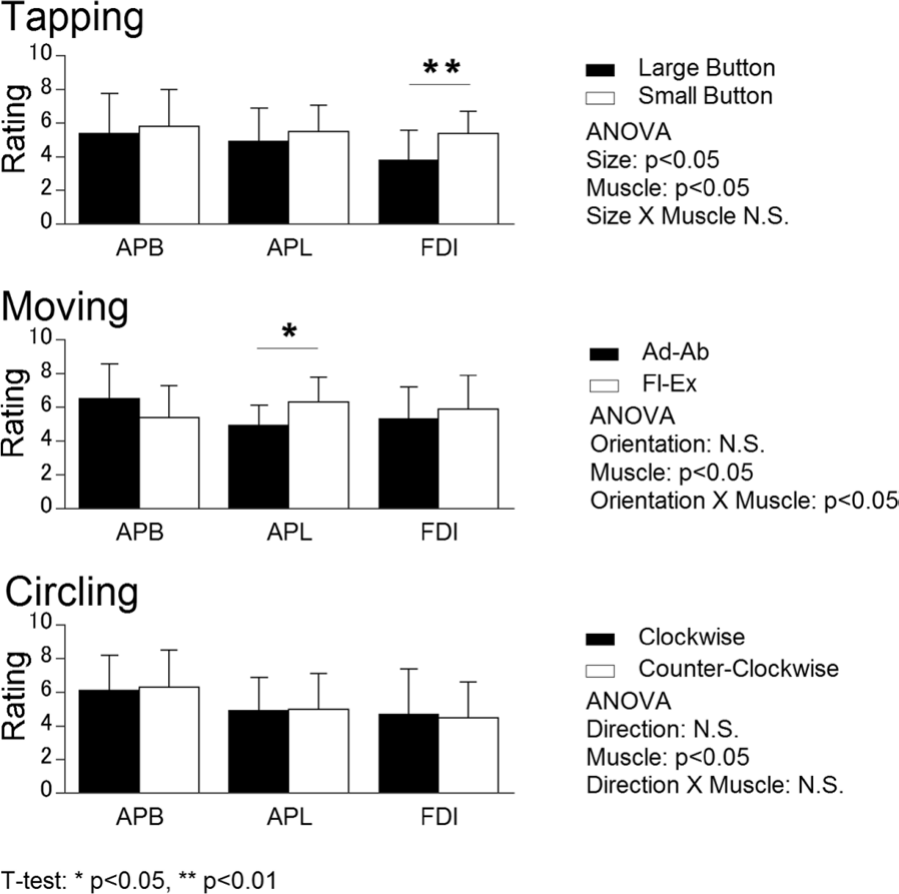
Perceived exertion rating in three tasks (n = 20). Values indicate mean ± SD

In terms of fatigue time, that in the larger button task was significantly longer than in the smaller button task for both fixed and max speed subtasks (Table 2). As for tapping speed, that in the larger button task was also significantly quicker than in the smaller button task (Table 2). Two-way ANOVA revealed significant main effects for button size and muscle (Fig. 3) in perceived exertion evaluation. Compared to the larger button task, the rating of FDI was significantly higher in the small button task (Fig. 3). The contraction time and iEMG of FDI significantly increased from the small button task to the large button task, but no statistically suggestive variations were detected in the other two muscles (Table 3).

**Table 2:** Comparison of thumb performance between tasks

	Tapping		Moving		Circling	
	Large	Small	Ad–Ab	Fl–Ex	Clockwise	C-Clockwise
Fatigue time (S)						
Fixed Speed	129.2 ± 43.1	77.8 ± 28.7**	74.7 ± 31.3	42.5 ± 15.3**	54.4 ± 22.1	52.2 ± 22.4
Max Speed	32.6 ± 12.3	24.8 ± 9.2**	22.7 ± 6.5	19.6 ± 11.5	23.8 ± 8.7	23.1 ± 10.3
Tapping Speed (tap/min)	199.8 ± 42.4	172.2 ± 31.4**	100.2 ± 20.8	88.2 ± 16.3**	96.0 ± 16.5	96.6 ± 14.8

Values indicate mean ± SD

T-test: * p < 0.05, ** p < 0.01

**Table 3:** EMG comparison in tapping task

	iEMG/S		Contraction time		iEMG	
	Large	Small	Large	Small	Large	Small
Fixed speed						
APB	1.12 ± 0.14	0.89 ± 0.09	0.92 ± 0.10	1.14 ± 0.12	0.97 ± 0.15	1.05 ± 0.16
APL	1.08 ± 0.09	0.95 ± 0.08	0.95 ± 0.11	1.15 ± 0.15	1.11 ± 0.13	1.22 ± 0.18
FDI	0.95 ± 0.12	1.10 ± 0.12	0.84 ± 0.09	1.42 ± 0.18*	1.12 ± 0.14	1.56 ± 0.23**
Max speed						
APB	0.96 ± 0.09	0.85 ± 0.08	1.01 ± 0.09	1.08 ± 0.09	0.99 ± 0.14	1.12 ± 0.15
APL	0.92 ± 0.08	1.10 ± 0.91	1.05 ± 0.93	0.95 ± 0.08	1.13 ± 0.15	1.22 ± 1.17
FDI	0.91 ± 0.09	1.21 ± 0.12	0.94 ± 0.14	1.34 ± 0.18**	0.92 ± 0.15	1.67 ± 0.31**

Values indicate percentage of reference values (mean ± SE)

Wilcoxon signed-rank test: * p < 0.05, ** p < 0.01

### Moving section

The fatigue time in flexion–extension task was shorter than that in ad–abduction task for the fixed speed task (Table 2). In terms of tapping speed, participants tapped significantly more quickly in the ad–abduction task than in the t flexion–extension ask (Table 2). As for perceived exertion, two-way ANOVA detected a significant main effect for muscle, while no significant result was obtained for orientation (Fig. 3). However, the interaction of muscle and orientation was revealed significant main effect (Fig. 3). In addition, the perceived exertion rating of APL was significantly higher in the flexion–extension task than in the ad–abduction task, while no significant variation was detected in the other two muscles (Fig. 3). From the ad–abduction task to the flexion–extension task, the contraction time and iEMG of APL significantly increased in both fixed and max speed subtasks, where those of FDI significantly increased only in the max speed subtask (Table 4). Meanwhile, no significant changes were found in APB for both fixed and max speed tasks (Table 4).

**Table 4:** EMG comparison in moving task

	iEMG/S		Contraction time		iEMG	
	Large	Small	Large	Small	Large	Small
Fixed speed						
APB	1.19 ± 0.14	1.11 ± 0.09	0.94 ± 0.12	1.07 ± 0.14	0.96 ± 0.17	1.19 ± 0.18
APL	1.18 ± 0.09	1.24 ± 0.08	0.97 ± 0.11	1.45 ± 0.27**	1.13 ± 0.18	1.78 ± 0.38**
FDI	1.11 ± 0.12	1.21 ± 0.12	0.96 ± 0.09	1.12 ± 0.14	1.22 ± 0.19	1.17 ± 0.16
Max speed						
APB	0.86 ± 0.09	0.86 ± 0.08	0.98 ± 0.10	1.12 ± 0.09	0.89 ± 0.17	1.05 ± 0.21
APL	1.3 ± 0.09	1.21 ± 1.10	0.88 ± 0.90	1.44 ± 0.26**	1.20 ± 0.16	1.76 ± 0.41**
FDI	0.84 ± 0.10	0.96 ± 0.11	0.91 ± 0.10	1.24 ± 0.24*	1.11 ± 0.15	1.48 ± 0.34**

Values indicate percentage of reference values (mean ± SE)

Wilcoxon signed-rank test: * p < 0.05, ** p < 0.01

### Circling section

The fatigue time and tapping speed demonstrated no significant changes among all three tested muscles (Table 2). A significant main effect of muscle was revealed in perceived exertion, but no other significant effects were found in direction, or in the interaction of direction and muscle (Fig. 3). None of statistically suggestive results of EMG indexes in clockwise and counter-clockwise tasks were exhibited.

## Discussion

### Tapping section

As the results show (Table 2), the elderly participants tapped significantly slower for the small button task, and also developed fatigue feeling significantly more rapidly. In the evaluation of perceived exertion, that of FDI was the only one showing increased perceived exertion when the task shifted from “large button” to “small button” (Fig. 3). This is in accordance with the EMG results of this muscle. That is the muscle activity of FDI significantly increased when the thumb shifted from the large button task to the small button one, whereas no statistically significant changes were found in the other two tested muscles (Table 3).

**Fig. 4:**
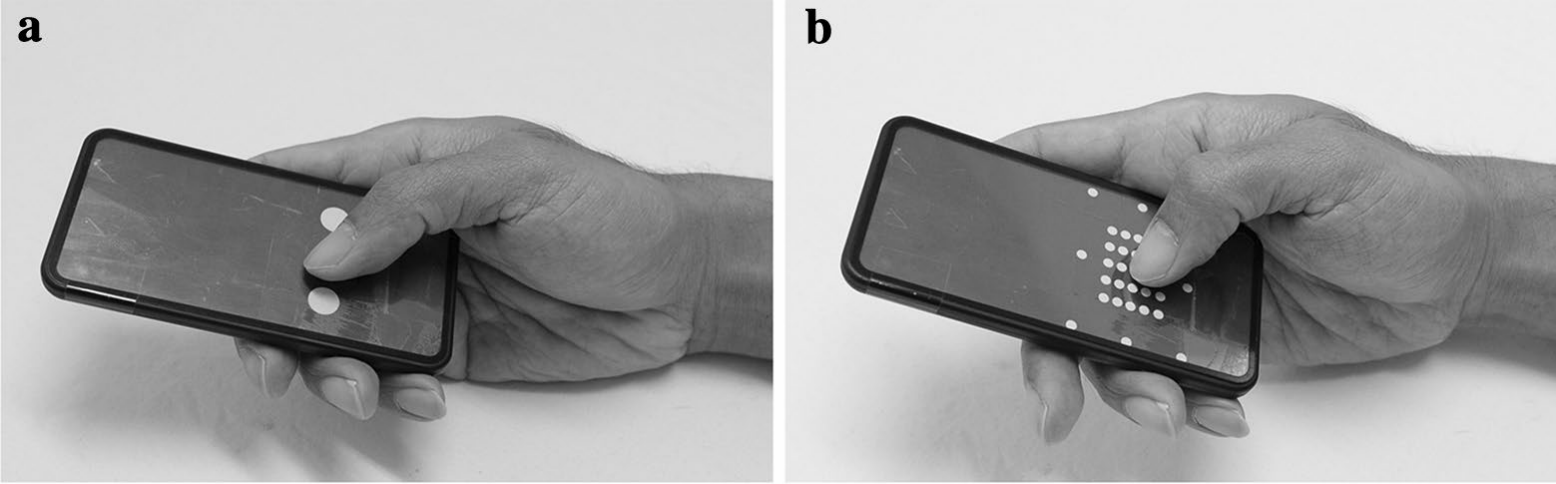
Tapping postures in tapping task (**a** large button, **b** small button)

These findings are in agreement with those found for young participants in the same experiments carried out by Xiong and Muraki (2014). According to the result, the assumption is validated that button size has significant effects on not only human performance but also physical work on smartphone touchscreens. That is, compared with large button size, small buttons greatly affect thumb performance, physical demand on thumb muscles, and thumb muscle activity. While others may explain this from a vision capability perspective, since smaller buttons are difficult to find due to the reduced visual acuity of elderly people, our study explains it from a thumb posture perspective. In the experiment, the thumbs of participants tended to adopt into a vertical posture in the small button task rather in the large button task (Fig. 4). The reason causing this adoption is considered that the contact area between the thumb tip and the screen is required to be reduced in order to tap a small target. Thus, to reduce the contact area, the participants had to raise their thumbs and maintain them in a vertical posture. By doing this, the accuracy of target selection could be maintained (Park and Han 2010). In addition, the position of the index finger also was important for tapping of the buttons. As Fig. 6 shows, the index finger placed underneath the mock-up acted as a significant grip finger working together with the thumb to retain the phone mock-up in the first web space. A previous study found that the muscle activity of FDI would increase when the thumb and index finger worked together to perform a precision grip (Anson et al. 2002). As Table 4 shows, FDI is the only muscle that has significantly longer contraction time for the small button task than for the large button task. This eventually caused the iEMG (overall muscle effort) of this muscle to be increased. This means that elderly people may place more demand on the FDI compared with other muscles to maintain a vertical posture of the thumb, in order to tap small touch buttons. Thus, the use of small buttons should be minimised to reduce the muscle effort, especially for FDI.

Apart from muscle activity in the thumb, another significant finding was that a small touch button has a greater impact on thumb performance in the elderly. Compared with a larger button, a smaller button is physically harder to locate. Thus, to maintain an acceptable accuracy of target selection, the elderly participants made more effort to retain a vertical thumb posture. In this case, performing a precision grip for performing the touchscreen operating tasks became a challenge for the elderly subjects with their decreased general hand functions (Carmeli et al. 2003; Ranganathan et al. 2001). Since general hand function is significantly decreased with age, the tapping pace could not be maintained by elderly users (Ranganathan et al. 2001). For this reason, elderly people are less likely than young users to retain a vertical thumb posture that could ensure tapping precision, despite making greater effort to do so. Therefore, smaller buttons were more difficult than larger buttons for the elderly to operate, the tapping speed was reduced, and fatigue appeared earlier as a result.

In addition, the FDI is a muscle providing distally and ulnarly directed force for the first metacarpal bone in the thumb (Brand and Hollister 1993). Thus, the elderly may increase the effort in this muscle to maintain the direction and stability of the thumb in the tapping of touch buttons. Another study found that the muscle activity of FDI increased with increasing neural activity of ulnar neuropathy in the wrist (Kim 2011). Their study further proposed that smaller buttons not only increase muscle disorders in the thumb by over-contraction, but also may increase the possibility of developing neural disorders in the operating hand and forearm. To sum up, owing to the increased effort demands on FDI, the thumb performance of elderly users upon tapping smaller touch buttons on a smartphone touchscreen decreased, while increasing the risk of physiological disorders in the operating thumb and hand.

### Moving section

In the results of tapping speed, the participants tapped significantly more rapidly in the ad–abduction task than in the flexion–extension task. For fatigue time, the fixed speed subtask showed that participants developed fatigue more rapidly in flexion–extension than in the ad–abduction task, although no significant variation was shown in the max speed subtask (Table 2). In the evaluation of perceived exertion, the APL was rated as the only muscle exhibiting greater perceived exertion when the task shifted from ad–abduction to flexion–extension. The same tendency of APL was also found in the EMG results (Table 4).

It is clear that the fatigue time and tapping speed of elderly participants decreased in the flexion–extension task, even though decreased fatigue time was only previously found for the young (Xiong and Muraki, 2014). However, APL was rated as the only muscle having increased perceived exertion when the task changed from ad–abduction to flexion–extension orientation movement. In addition, the EMG results showed that the muscle workload of APL increased in both fixed and max speed subtasks when the thumb shifted from the ad–abduction to the flexion–extension task, while that of FDI increased only in the max speed subtask. As for APB, no statistically suggestive results were found in both the perceived exertion evaluation and the EMG analysis. In the same experiments by Xiong and Muraki (2014), it was found that the perceived exertion and muscle workload of APB increased in ad–abduction movement, and those of FDI increased in flexion–extension movement, while no statistically suggestive changes were shown in the EMG analysis of APL.

**Fig. 5:**
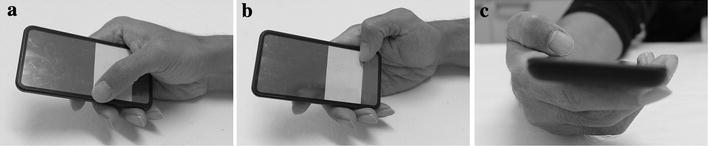
Moving postures in ad–abduction task (**a** *top-right* adduction corner, **b** *bottom-left* abduction corner, **c** grip posture of ad–abduction task)

**Fig. 6:**
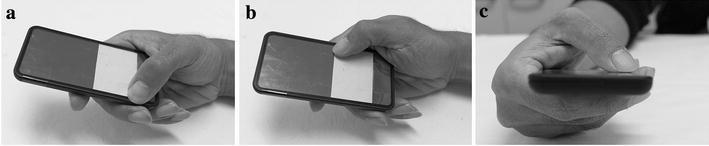
Moving postures in flexion–extension task (**a** distant extension corner, **b** proximal flexion corner, **c** grip posture of proximal flexion corner)

The reason for this is considered to be that flexion–extension movement caused the elderly to have a different workload distribution among the thumb muscles when operating a smartphone touchscreen compared with the young. In the experiment, the participants retained their thumbs within an oblique posture during the ad–abduction task (Fig. 5), but adopted a vertical posture when the task shifted to the flexion–extension task, especially in the bottom-right corner (Fig. 6). Compared with young participants, elderly participants had a 30 % decrease in grip force (Ranganathan et al. 2001). This may have caused the elderly participants to be less able to involve either FDI or APB in maintaining the grip force. Hence, for compensating the loss of grip force, and maintain the stability of the thumb, the elderly participants exerted more muscle effort in the APL. By doing so, the thumb performance in the flexion–extension task could be maintained. This is matched with the previous studies, which found that APL has a stabilizing function at the CMC joint and the basal side of the thumb (Li et al. 2008; Oudenaarde et al. 1995). Britto and Elliot (2002) also pointed out that the APL does not function to generate power in a grip, but works together with the APB as a thumb stabiliser. In addition, compared with young users (Xiong and Muraki 2014), the thumb performance of elderly participants (both fatigue time and tapping speed) was significantly poorer (Table 4). As stated above, the decreased general hand function of elderly people may be accounted for by their decreased thumb performance; the increased demand on APL is considered the main reason for the poorer performance of elderly people in the specified moving task. Owing to their decreased general hand function and grip force, elderly people tended to require greater effort in order to retain the thumb postures. Since retaining the thumb posture also required the thumb to be more stable, the demands on APL increased. As Fig. 3 shows, the perceived exertion of APL in the flexion–extension task significantly increased compared with that in the ad–abduction task, which shows that the physical demands on this muscle for the flexion–extension task are much greater. Since the general hand function decreased, the increased demands on APL actually caused difficulty of involving this muscle. As a result, the general thumb performance of elderly participants decreased.

Moreover, in addition to acting as a thumb stabiliser, the APL also works as an extensor in the thumb. According to Li et al. (2008) and Cooney et al. (1984), in spite of the same results of APL suggesting that this muscle may work as an abductor, actually, this muscle produces extension and supination rather than abduction at the CMC joint. As the EMG results show (Table 4), the contraction time of APL is significantly longer in the flexion–extension task than in the ad–abduction task, which eventually caused the muscle effort in the flexion–extension task to be greater. This is also considered as the reason that caused the APL to develop fatigue more rapidly. For the increased muscle activity of FDI in the max speed subtask, it is considered that, since the task pressure was increased, the FDI was involved more in supporting maintenance of the thumb in vertical posture when tapping for the flexion–extension task (Xiong and Muraki 2014). Therefore, the elderly participants tended to involve APL more in maintaining the stability of the thumb when performing the flexion–extension task while the overall grip force declined. Practically speaking, elderly users seem to be at a higher risk of developing muscular disorders than their younger counterparts since they might move their thumbs more strenuously, crossing over the surface of the touchscreen, to compensate for their decreased visual acuity, muscle strength and motor skills (Macaluso and De Vito 2004; Tartarisco et al. 2012). This risk could become higher when the elderly adopt the thumb posture in order to perform flexion–extension movements. Therefore, the designs of user interfaces of touchscreen smartphones need to consider the movement characteristics of the thumb, rather than simply varying the sizes of touch targets or screens. Since performing flexion–extension tasks places increased demands on the APL, the thumb performance of elderly participants was decreased compared with that in performing ad–abduction task.

### Circling section

No statistically suggestive findings were obtained in this section. To perform the tasks of the circling section, the participants tapped all the four corners in the tested area, in two different directions: clockwise and counter-clockwise. This exposed the thumb to even more difficult and complicated movements and greater task pressure. Moreover, considering that the elderly tend to have decreased hand functions compared with the young (Carmeli et al. 2003; Ranganathan et al. 2001), it was expected that greater variations in perceived exertion evaluation and muscle activity would occur. However, no significant differences were found in thumb performance (Table 2), perceived exertion evaluation (Fig. 3) and EMG analysis in the comparison between these two circling directions. This means that circling direction does not affect the thumb performance of elderly participants in terms of using a smartphone touchscreen with right hand. This also accords with the findings of a previous study on young participants (Xiong and Muraki 2014), except that the overall thumb performance of elderly participants was poorer than that of the younger ones.

### Limitations

The insufficient experience of using a touchscreen smartphone on a daily base among the subjects is one limitation of this study. Owing to this, the holding postures or operating postures of the thumb may have been somewhat distorted. This could also have affected the distribution of muscle workload among the thumb muscles. In addition, other muscles (in hand, arm and shoulder) that may be involved in holding and operating of a smartphone were not included in this study. Thus, the effects of these muscles on the phone mock-up holding posture and thumb movements were not covered in this study. Another limitation is that the risks of potential injury and fatigue development were not covered in the present study. For addressing this issue that identifies the levels of injury risk and fatigue development in the thumb muscle, it is necessary to carry out the future studies, in which objective approaches are developed to determine the thresholds of injury risk and fatigue development, in the comparisons of muscle activity among the targeted muscles.

## Conclusions

In terms of smartphone operation, the button size tends to affect how elderly users perform thumb operating tasks on smartphone touchscreens. The muscle workload and perceived exertion of FDI increased when the thumb tapped smaller buttons (diameter: 3.0 mm) compared with larger buttons (diameter: 9.0 mm). This study regarded that increasing button size is the main reason that caused the thumb to develop fatigue more rapidly. Compared with ad–abduction, the flexion–extension orientation task showed rapid fatigue development and decreased tapping speed. Meanwhile, the muscle workload and perceived exertion of APL increased. This is considered as the main reason for the decreased thumb performance in the flexion–extension task. Furthermore, circling direction does not affect thumb performance on a smartphone touchscreen since no statistically suggestive results were obtained. Taking all this into account, this study recommends to minimise the use of small buttons and flexion–extension movements in the design of handheld device interfaces for aging users. This is to reduce the effort-related demands on FDI and APL that potentially causes the thumb to be less susceptible to fatigue and slow tapping.

Finally, the study suggests that the design of user interfaces for small touchscreen devices must minimise the negative effects of thumb posture adoption for performing flexion–extension movements on the screen. First of all, the placement of frequently used targets at the right and lower sides should be avoided or minimised (for right hand operation). In addition, the right part of the (English) keyboard should be raised and slightly left-slanted to reduce the frequency of flexion–extension movements. By doing this, the frequency of performing flexion–extension movements could be minimised to reduce the negative effects of adopting a vertical postures of the thumb.
